# Gem-Induced Cytoskeleton Remodeling Increases Cellular Migration of HTLV-1-Infected Cells, Formation of Infected-to-Target T-Cell Conjugates and Viral Transmission

**DOI:** 10.1371/journal.ppat.1003917

**Published:** 2014-02-27

**Authors:** Sébastien A. Chevalier, Jocelyn Turpin, Anne Cachat, Philippe V. Afonso, Antoine Gessain, John N. Brady, Cynthia A. Pise-Masison, Renaud Mahieux

**Affiliations:** 1 Equipe Oncogenèse Rétrovirale, Equipe labellisée “Ligue Nationale Contre le Cancer”, International Center for Research in Infectiology, INSERM U1111 - CNRS UMR5308, Ecole Normale Supérieure de Lyon, Université Lyon 1, Lyon, France; 2 Epidémiologie et Physiopathologie des Virus Oncogènes, CNRS UMR 3569, Pasteur Institute, Paris, France; 3 Virus Tumor Biology Section, Laboratory of Cellular Oncology, National Cancer Institute, National Institutes of Health, Bethesda, Maryland, United States of America; 4 Animal Models and Retroviral Vaccine Section, Vaccine Branch, CCR, National Cancer Institute, National Institutes of Health, Bethesda, Maryland, United States of America; University of Pennsylvania School of Medicine, United States of America

## Abstract

Efficient HTLV-1 viral transmission occurs through cell-to-cell contacts. The Tax viral transcriptional activator protein facilitates this process. Using a comparative transcriptomic analysis, we recently identified a series of genes up-regulated in HTLV-1 Tax expressing T-lymphocytes. We focused our attention towards genes that are important for cytoskeleton dynamic and thus may possibly modulate cell-to-cell contacts. We first demonstrate that Gem, a member of the small GTP-binding proteins within the Ras superfamily, is expressed both at the RNA and protein levels in Tax-expressing cells and in HTLV-1-infected cell lines. Using a series of ChIP assays, we show that Tax recruits CREB and CREB Binding Protein (CBP) onto a c-AMP Responsive Element (CRE) present in the *gem* promoter. This CRE sequence is required to drive Tax-activated *gem* transcription. Since Gem is involved in cytoskeleton remodeling, we investigated its role in infected cells motility. We show that Gem co-localizes with F-actin and is involved both in T-cell spontaneous cell migration as well as chemotaxis in the presence of SDF-1/CXCL12. Importantly, *gem* knock-down in HTLV-1-infected cells decreases cell migration and conjugate formation. Finally, we demonstrate that Gem plays an important role in cell-to-cell viral transmission.

## Author Summary

HTLV-1 was the first human oncoretrovirus to be discovered. Five to ten million people are infected, and 1–6% will develop either Adult T-cell Leukemia, or Tropical Spastic Paraparesis/HTLV-1 Associated Myelopathy (TSP/HAM). HTLV-1 infects primarily T-cells, but dendritic cells were also found to carry proviruses. Contrary to HIV-1, cell-free HTLV-1 viral particles are poorly infectious. Thus, efficient viral transmission relies on formation of virological synapses or formation and transfer of viral biofilm-like structures. The Tax viral transactivator plays a key role in both modes of transmission. Using transcriptomic analyses, we recently identified cellular genes that are deregulated following Tax expression in T-cells. We focused our attention on genes that are important for cell architecture and are thus likely to modulate cell-to-cell contacts and motility. We found that Gem was highly upregulated both at the RNA and protein levels in Tax-expressing cells and HTLV-1-infected cell lines. We further show that Tax binds cellular co-activators and transcription factor and activates transcription from the *gem* promoter. We demonstrated that Gem is involved in cellular migration of HTLV-1-infected cells. Importantly, *gem* knockdown decreases the rate of HTLV-1-infected cell migration and cell-to-cell conjugate formation. We also show that Gem plays an important role in HTLV-1 transmission through cell-to-cell contacts, the most efficient mode of viral infection.

## Introduction

Five to 10 million people are infected with the HTLV-1 retrovirus (Human T-cell Leukemia Virus Type 1) worldwide [1]; and 1–6% of infected people will develop either Adult T-cell Leukemia (ATL) [2], a malignant lymphoproliferation of mature activated T-cells, or inflammatory disorders, such as Tropical Spastic Paraparesis/HTLV-1 Associated Myelopathy (TSP/HAM) [3], [4].

The long period of clinical latency between primo-infection and ATL outcome suggests that the pathogenesis is a complex multistep process [5]. The exact mechanism by which infected individuals develop ATL is still debated, although the Tax viral oncoprotein has clearly been associated with cell transformation. Indeed, Tax not only activates viral expression but it also triggers a wide range of cell-signaling pathways, reprograms cell cycle, interferes with control checkpoints and inhibits DNA repair [6], [7]. Tax immortalizes/transforms T-lymphocytes *in vivo* and *in vitro* [8] and promotes tumors in transgenic mice [9].

In contrast to HIV-1, HTLV-1-infected cells produce few free viral particles [10]. Therefore, efficient transmission of HTLV-1 from infected to uninfected T-cells relies on cell-to-cell contacts, both *in vitro* and likely *in vivo* [7], [11]. Transmission of HTLV-1 particles to an uninfected T-lymphocyte occurs through two mutually non-exclusive models: (i) formation of a virological synapse between an infected lymphocyte and an un-infected lymphocyte (involving cytoskeleton reorganization and reorientation of the microtubule-organizing center (MTOC) in the infected T-lymphocyte) [12] or (ii) formation and transfer of a viral biofilm-like structure [13]. In the first situation, Tax promotes MTOC polarization and is found at the cell-to-cell junction and around the MTOC, together with the viral gag p19 protein [14], [15], while viral particles might be transferred within the synaptic cleft [16]. In the biofilm model, HTLV-1 particles are stored and attached together on the outer cell surface in a virus-induced extracellular matrix composed of collagen, agrin and cellular linker proteins (tetherin and galactin3). During cell-to-cell contacts, these embedded extracellular virions spread and infect target cells. Tax plays also an important role by increasing the production of collagen, a component of this matrix [13]. Whether other cellular genes are targeted by Tax and enhance cell-to-cell transfer is currently a matter of investigation.

Using GeneChip Affymetrix technology, we recently identified a series of genes up-regulated in T-lymphocytes transduced by Tax-expressing lentivirus [17]. Among these genes, we focused our attention towards those that are important for cytoskeleton dynamic and thus possibly modulate cell-to-cell contacts. Gem, a member of the small GTP-binding proteins within the Ras superfamily [18], was found to be up-regulated following Tax expression [17]. Gem, together with Rad, Rem, and Rem2, form a subfamily of atypical proteins of the Ras family, often termed RGK (for Rad and Kir/Gem). Indeed, these proteins present significant variations, compared with other Ras family proteins in the regions involved in binding phosphate moieties of guanine nucleotides. The conserved threonine residue (T35) of the switch 1 region (often referred to as the effector region) that coordinates magnesium ion complexing the *beta* and g*amm*a phosphates of GTP (Mg-GDP/GTP binding) is absent, and the DXXG region of switch 2 which contributes to nucleotide binding and GTPase catalysis is profoundly modified to DXWE. As a consequence, Gem markedly prefers GDP over GTP, and more importantly, Gem has an undetectable intrinsic GTPase activity [18]–[20].

Activation or deregulation of Ras signaling pathways causes cell growth, differentiation and survival and can ultimately lead to oncogenesis and cancer [21]. Gem expression is induced in human peripheral blood T-cells by mitogens such as PHA or PMA [22] and promotes cell shape remodeling by restructuring actin cytoskeleton and microtubule networks. In epithelial cells, Gem induces cell elongation, with disappearance of actin stress fibers, loss of focal adhesion and enhancement of the actin cortical network [23]. Using a library of randomized hybrid ribozymes, Gem was identified as an actor in cell invasiveness, a process that is essential for tumor metastasis [24]. In neuroblastoma cells, Gem antagonizes Rho kinase-induced neurite retraction and morphological differentiation [18]. Therefore, we hypothesize that Gem could play an essential role in HTLV-1 transmission.

We demonstrated here that Gem is highly expressed at the RNA and protein levels in Tax-expressing cells and HTLV-1-infected cell lines. We further delineated the mechanism of Tax-induced *gem* transactivation and show that Tax recruits CREB and the CBP co-activator onto a CRE sequence present on the *gem* promoter. Gem protein co-localizes with F-actin and is involved in spontaneous cell migration and chemotaxis. Importantly, we observed that *gem* knock-down decreases the rate of HTLV-1-infected cell migration and conjugate formation. Most importantly, we demonstrated that Gem expression in HTLV-1-infected cells is involved in viral transmission, from infected cells to target cells through enhanced cell-to-cell contacts.

## Results

### *Gem mRNA* and Gem protein levels are up-regulated in Tax-expressing and HTLV-1-infected cells

Using transcriptional profiles analyses, we recently identified genes that are up-regulated in T-lymphocytes transduced by Tax lentivirus [17]. In order to identify genes that could play a role in cell transmission, we focused our retrospective analysis on genes that are involved in cytoskeleton remodeling. Interestingly, *gem* mRNA was strongly up-regulated (28 fold) in Tax expressing vs. control cells. We also performed a database search on HTLV gene expression profiles and uncovered that *gem* up-regulation was also listed in previous studies that used high-throughput approaches on Tax expressing lymphocytes, HTLV-1-infected cells lines or HTLV-1 immortalized cells [25]–[27] (Table S1). These observations led us to investigate in more details the mechanisms and biological consequences of Tax-induced *gem* transactivation.

**Figure 1:**
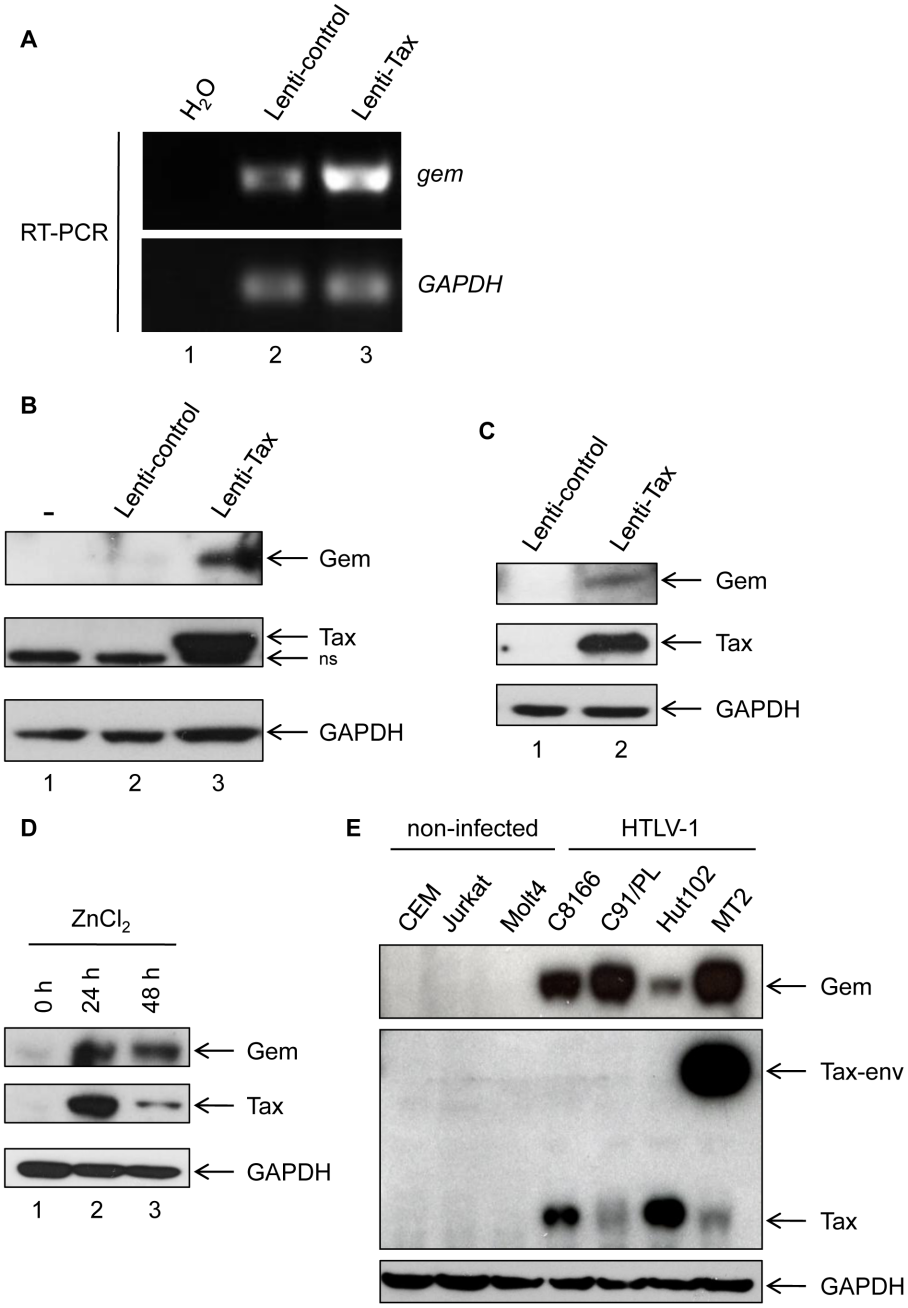
Gem is overexpressed in T- and non-T-Tax-expressing cells as well as in HTLV-1 infected cells. (A): Total RNA was extracted from 293T cells transduced with Lenti-control (lane 2) or Lenti-Tax (lane 3) and RT-PCR was performed using *gem* or *GADPH* specific primers. Lane 1 is a control of extraction. (B, C): Western blot analyses were performed with 70 µg of proteins from (B) 293T or (C) MOLT4 cells transduced by Lenti-control or Lenti-Tax vectors (72 h post-transduction). (D): JPX-9 cells were grown for 24 h or 48 h in the presence of ZnCl_2_ (120 µM). Western blot analyses were performed with 70 µg of JPX-9 cellular extracts. (E): Western blot analyses were performed with 70 µg of cellular extracts obtained from non-infected (CEM, Jurkat and MOLT4) or HTLV-1-infected (C8166, C91/PL, Hut102 and MT2) T-lymphocytes. (B, C, D, E): Membranes were probed with anti-Gem (1∶2,000) and anti-GAPDH (1∶1,000) or anti-Tax Tab 172 (1∶500) antibodies.

We first confirmed the microarray analyses by performing RT-PCR experiments and demonstrated that *gem* expression is strongly up-regulated in 293T cells transduced with Tax lentiviral particles but not in cells transduced with a control lentivirus (Figure 1A lane 2 vs. 3). Gem protein was also strongly overexpressed in 293T and MOLT4 cells transduced with Tax-expressing lentivirus, while it was undetectable in the absence of Tax expression (Figure 1B and 1C). As controls, Tax and GAPDH protein levels were evaluated by western blot.

We also used the Tax inducible JPX-9 T-cell line to confirm the effect of Tax on Gem expression (Figure 1D). Tax expression was induced by addition of ZnCl_2_ in the culture media for 24 h or 48 h. Interestingly, in that lymphocytic cell line, Gem expression also follows Tax induction. We then assessed Gem expression in a series of HTLV-1-infected cell lines (C8166, C91/PL, Hut102 and MT2). All cell lines tested showed a high level of Gem protein expression, while in non-infected T-lymphocytes (CEM, Jurkat and MOLT4) Gem expression was not detectable (Figure 1E). As controls, Tax and GAPDH protein levels were evaluated by western blot. Altogether, these results demonstrate that Tax expression is sufficient to induce Gem expression both at RNA and protein levels in different cell types.

### Tax recruits CREB and CBP onto the Gem promoter

**Figure 2:**
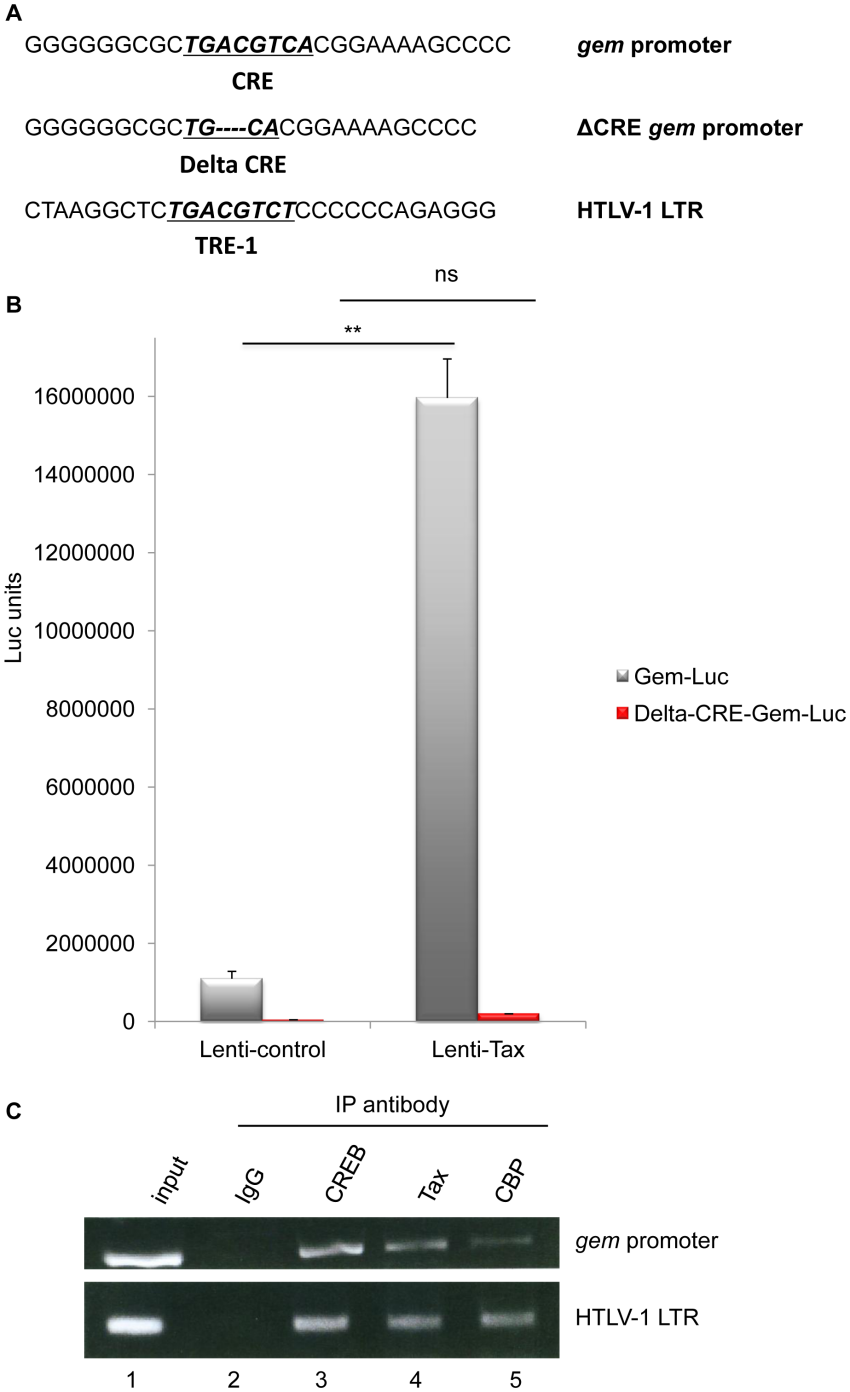
Together with CREB and CBP, Tax is present on the Gem promoter and activates its transcription. (A): Schematic representation of the c-AMP Responsive Element (CRE) and Tax Responsive Element (TRE) sequences present in the *gem* promoter and in the HTLV-1 viral promoter, respectively. The core CRE sequence is underlined and in bold. ΔCRE corresponds to deletion of the 4 central nucleotides of the core CRE sequence (B): 293T cells were transduced with Lenti-control or Lenti-Tax particles. Forty-eight hours later, transduced cells were transfected with 500 ng of Gem-luc or Δ-CRE-Gem-luc reporter plasmids, 10 ng of phRG-TK (Promega), and DNA amount was adjusted with backbone vector. Transfection results were normalized to Renilla activity. The results represent the average of at least 3 independent experiments. **: significantly different, p = 4.10^−4^, ANOVA and Tukey post-hoc test, ns = non significant (C): Chromatin immunoprecipitation experiments were performed with C8166 (HTLV-1-infected) cells. ChIP assay was carried out using 10 µg of Tax (Tab 172), CREB or CBP antibody as previously described [44], [45]. PCR was performed using primers specific for the HTLV-1 LTR or *gem* promoter.

The sequence of *gem* promoter contains a cAMP Responsive Element (CRE) [28]. This sequence is very similar to the Tax Responsive Elements (TRE) present in the U3 region of the 5′ viral Long Terminal Repeat (Figure 2A).

To determine whether the CRE sequence in the *gem* promoter is required for Tax-mediated transactivation, we compared transactivation levels of reporter plasmids encompassing either the full-length *gem* promoter (Gem-luc) or the CRE-deleted promoter (ΔCRE-Gem-luc) (Figure 2A). Reporter plasmids were transfected in 293T cells previously transduced with lentiviral particles allowing Tax expression. In contrast to the control vector, Tax significantly activated transcription from the wild-type *gem* promoter (p = 4.10^−4^, ANOVA and Tukey post-hoc test), but not from the ΔCRE promoter, thus indicating that the CRE sequence is indeed necessary for Tax-mediated transactivation (Figure 2B).

It is well established that Tax activates the viral promoter (5′LTR) through its ability to bind CREB and its co-activators CREB Binding Protein (CBP) and p300 [29]. To determine whether Tax activates transcription from the *gem* promoter through a similar mechanism, we performed Chromatin immunoprecipitation assays (ChIP) using extracts from C8166 cells (Tax-expressing cells). This allowed us to demonstrate that Tax, CREB and CBP were present on the *gem* promoter (Figure 2C lanes 3, 4 and 5). The HTLV-1 LTR (lower panel) was used as control for complex binding.

These results indicate that *gem* transcription results from a direct interaction between Tax, CREB and CBP on the CRE sequence present in the *gem* promoter.

### Gem colocalizes with actin but not with Tax and promotes changes in cell morphology

**Figure 3:**
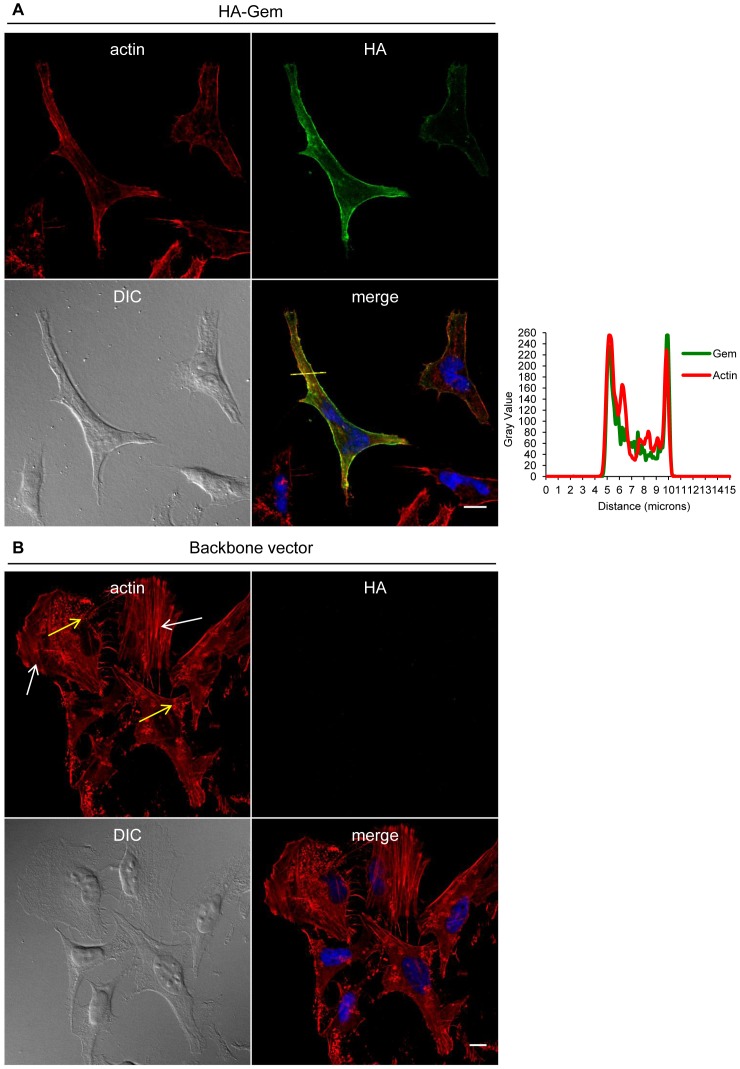
Gem expression induces actin re-localization. (A, B): HeLa cells were transfected with 400 ng of (A): HA-Gem, (B): backbone vector. (A, B): Twenty-four hours later, cells were fixed and stained with anti-HA antibody, followed by anti-rabbit-IgG FITC-conjugated antibody. Actin was stained with Rhodamine-Phalloïdin toxin (R415 300U, Life Technologies). Nucleic acids were stained with DAPI-containing mounting medium (DAPI Fluormount G, Southern biotech). Cells were visualized and acquired with a spectral Leica SP5 confocal microscope. (B): White and yellow arrows represent actin stress fibers and focal adhesion points, respectively. (A): Two-dimensional graph representing pixel intensities (gray level) along a 15 micrometers line (see yellow line on the merged image) was plotted. Green line displays HA-Gem signal intensity and red line displays actin signal intensity (Rhodamine-Phalloïdin).

**Figure 4:**
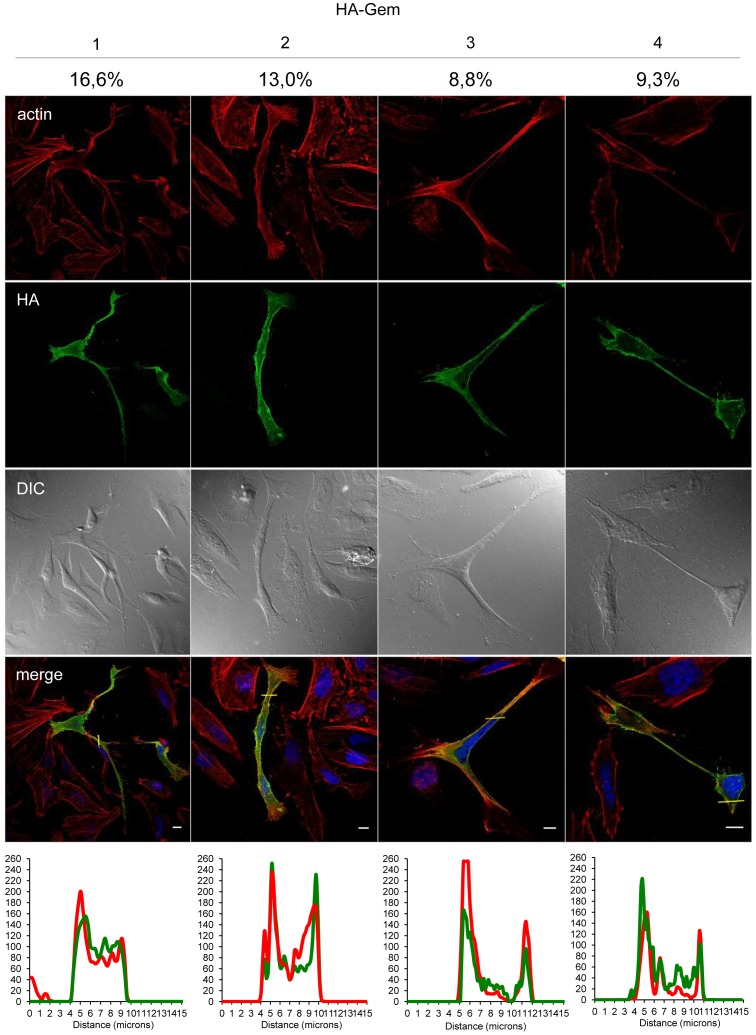
Gem expression induces morphological changes. HeLa cells were transfected with 400-Gem. Twenty-four hours later, cells were fixed and stained with anti-HA antibody, followed by anti-rabbit-IgG FITC-conjugated antibody. Actin was stained with Rhodamine-Phalloïdin toxin (R415 300U, Life Technologies). Nucleic acids were stained with DAPI-containing mounting medium (DAPI Fluormount G, Southern biotech). Cells were visualized and acquired with a spectral Leica SP5 confocal microscope. The percentage of each described phenotype (“1”, “2” “3”, “4”) was counted manually. Two-dimensional graphs representing pixel intensities (gray level) along a 15 micrometers line (see yellow line on the merged image) were plotted. Green line displays HA-Gem signal intensity and red line displays actin signal intensity (Rhodamine-Phalloïdin).

**Figure 5:**
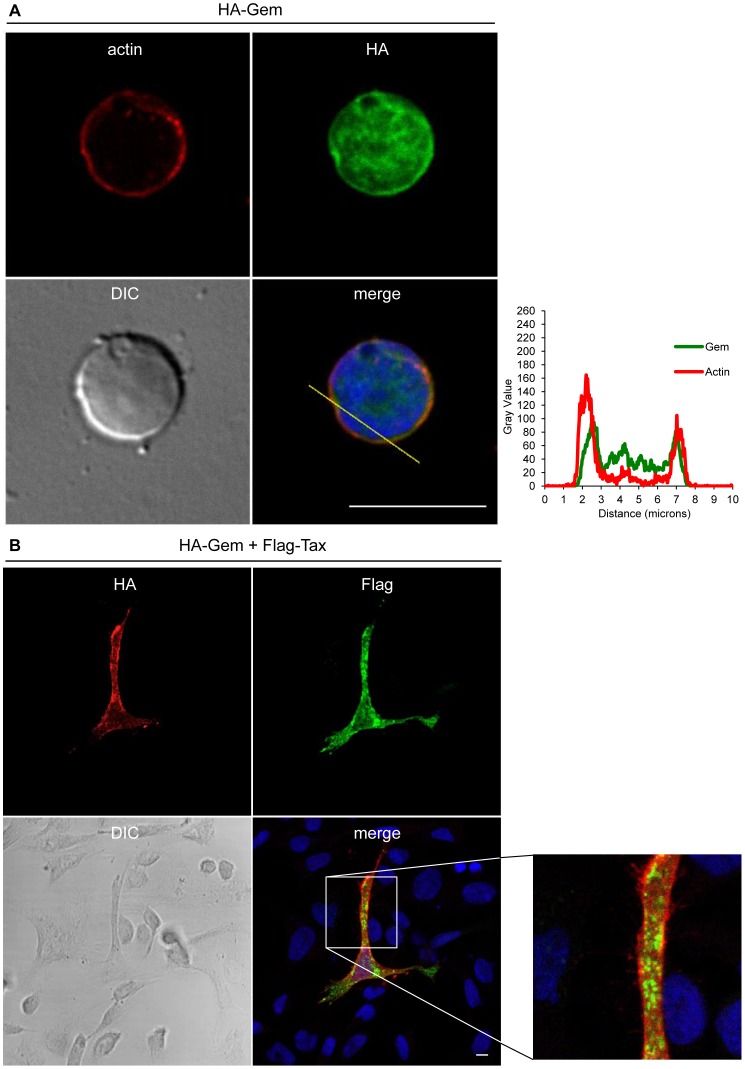
Gem colocalizes with actin but not with Tax. (A): PBLs were electroporated with 5 µg of HA-Gem and forty-eight hours later cells were processed as in (B). (B): HeLa cells were transfected with 400 ng of HA-Gem, or (B): co-transfected with 400 ng of HA-Gem and 400 ng of Flag-Tax. DNA amount was adjusted with backbone vector. Twenty-four hours later, cells were fixed and stained with anti-HA or anti-Flag antibody, followed by anti-rabbit-IgG FITC-conjugated or anti-mouse-IgG CY3-conjugated antibody, respectively. Actin was stained with Rhodamine-Phalloïdin toxin (R415 300U, Life Technologies). Nucleic acids were stained with DAPI-containing mounting medium (DAPI Fluormount G, Southern biotech). Cells were visualized and acquired with a spectral Leica SP5 confocal microscope. (A): Two-dimensional graphs representing pixel intensities (gray level) along a 10 micrometers line (see yellow line on the merged image) were plotted. Green line displays HA-Gem signal intensity and red line displays actin signal intensity (Rhodamine-Phalloïdin).

Gem was previously reported to be involved in cell cytoskeleton remodeling [23]. To determine whether Gem colocalizes with F-actin, a Gem-expressing vector was transfected and immunofluorescence experiments were performed in different cell types (Figures 3, Figure 4 and Figure 5). Gem promoted cell elongation, with a disappearance of actin stress fibers and a loss of focal adhesion points (Figure 3A), which were still present in cells transfected with backbone vector (Figure 3B, see actin stress fiber (white arrows) and focal adhesion points (yellow arrows)). Importantly, Gem colocalized with cortical actin filaments in non-T and in T cells (Figure 3A, Figure 4 and Figure 5). Gem-expressing cells presented various shapes and lengths with very long extensions as well as membrane protrusions (Figure 4). As previously described for N1E-115 mouse neuroblastoma cells and NIH3T3 cells [30], Gem expression induced unusual dendritic morphology with (Figure 4, right panel “4”) or without (left panel “1”) a large “synaptic bouton” morphology. Flattened bipolar (“2”) or multipolar (“3”) cells, notably described as an intermediate phenotype in N1E-115 cells expressing a mutated MLC (18D, 19D) were also observed.

Due to its role in cytoskeleton reorganization and reorientation of the MTOC during the formation of the virological synapse [12], [14], [15], we hypothesized that Tax could also colocalize with Gem in the actin-rich membrane protrusions. A Flag-Tax plasmid was transfected together with a HA-Gem plasmid and immunofluorescence experiments were performed. Tax and Gem colocalization could not be observed (Figure 5B). Even at high magnification (Figure 5B), Tax cytoplasmic speckles (visible in green) did not merge with Gem signal (visible in red). This result was also confirmed by co-immunoprecipitation experiments, which did not demonstrate any interaction between Tax and Gem (data not shown).

### Gem is directly implicated in the migration of HTLV-1-infected cells

Cell mobility follows a perpetual reorganization of the actin cytoskeleton, where monomers of actin polymerize to form fibers [31]. A previous report demonstrated that HTLV-1-infected T-lymphocytes have increased mobility [32], which could be beneficial for cell-to-cell viral transmission by increasing the probability of encountering uninfected target cells. Moreover, HTLV-1-infected T-lymphocytes induce blood brain barrier (BBB) disruption. This allows infected cells to enter into the central nervous system (CNS) [33]. Because Gem promotes reorganization of the cytoskeleton, we hypothesized that it could play a role in the observed enhanced cell motility of HTLV-1-infected cells.

**Figure 6:**
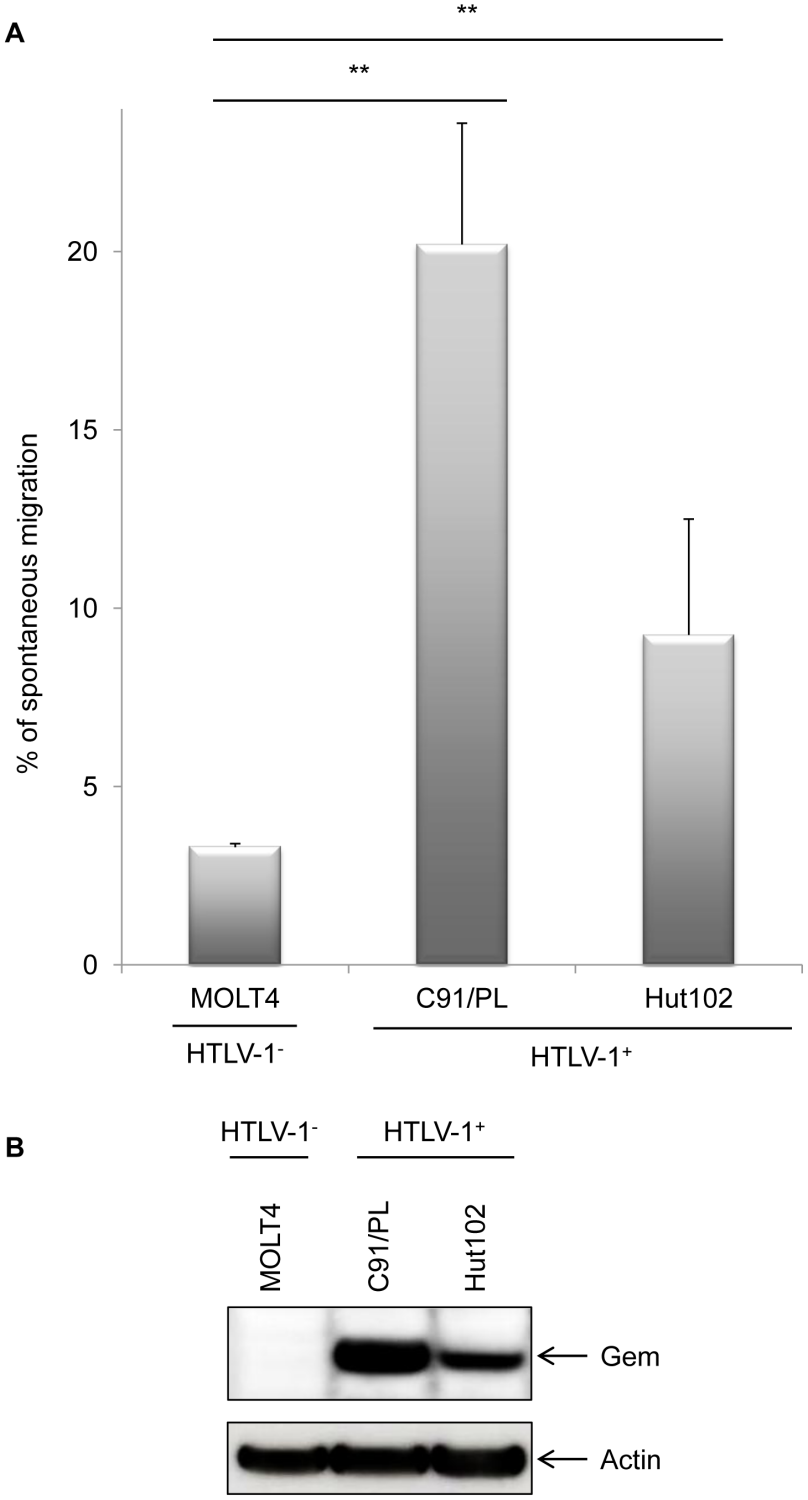
Gem expression level is correlated to cell migration. (A): Non-infected (MOLT4) or HTLV-1-infected (C91/PL and Hut102) T-lymphocytes (5×10^5^) were loaded on a 5 µm permeable Transwell filter. Twenty-four hours later, cell migration was quantified by flow cytometry (flow cytometer Facscalibur4c+HTS (BD biosciences)). (B): Western blot analyses were performed on 70 µg of cellular extracts from non-infected (MOLT4) or HTLV-1-infected (C91/PL and Hut102) T-lymphocytes. Membranes were probed with anti-Gem (1∶2,000) or anti-β-actin (1∶40,000) antibody (Sigma). **: significantly different, p<0.001, ANOVA and Dunnet post-hoc test.

We therefore performed a series of Transwell migration assays. The ability of cells to transfer from the upper chamber to the lower chamber of a Transwell permeable filter was measured and the percentage of migrating cells was then quantified by flow cytometry. We first observed that C91/PL and Hut102 (HTLV-1-infected cell lines) had a higher mobility than non-infected MOLT4 T-cells (Figure 6A, p<0.001, ANOVA and Dunnet post-hoc test). Interestingly, the ability of HTLV-1 cells to migrate seemed to correlate with Gem expression levels (Figure 6B), suggesting that HTLV-1-induced migration increase was associated with Tax-induced Gem overexpression.

**Figure 7:**
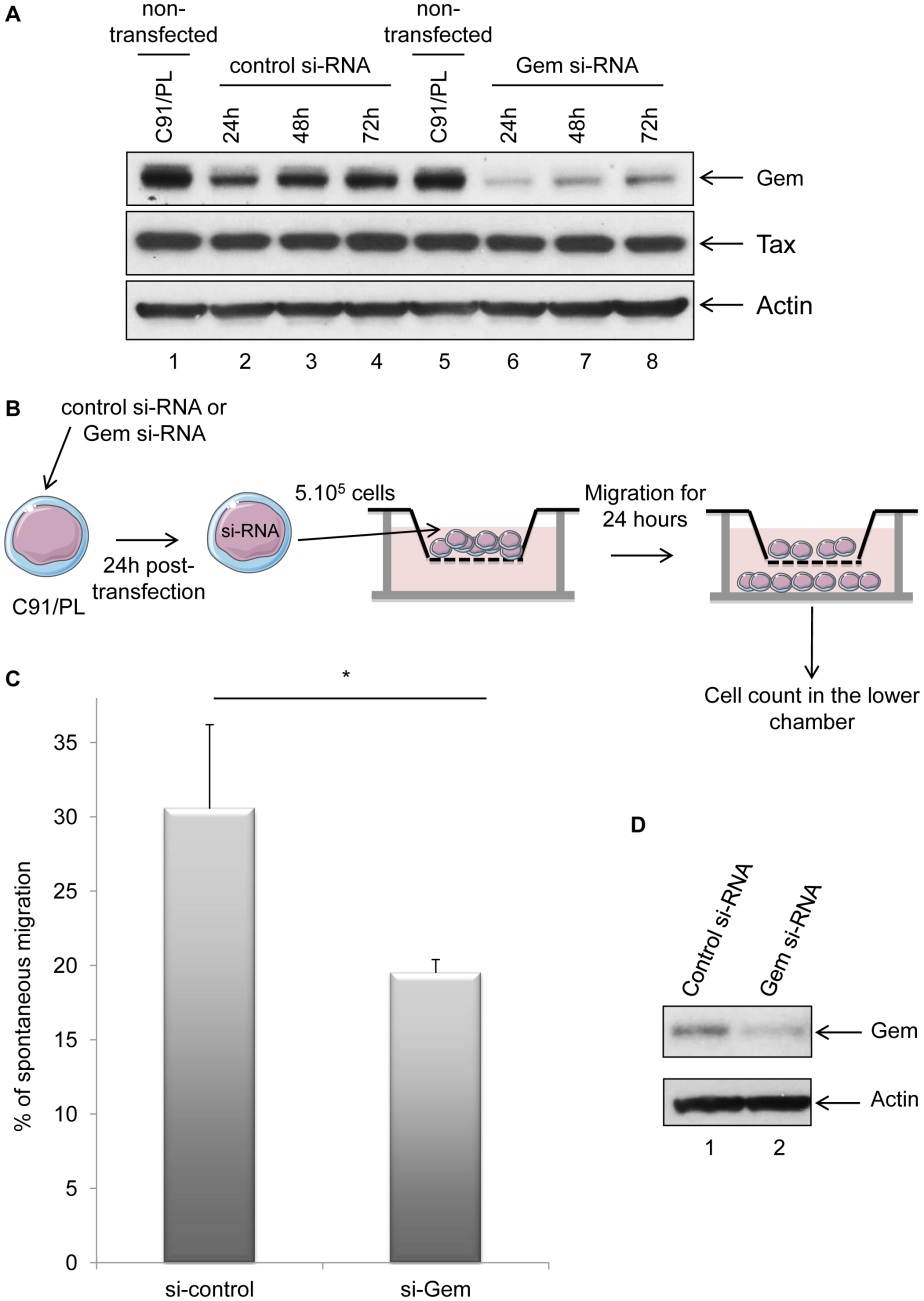
Gem is involved in HTLV-1-infected cell migration. (A): Western blot analyses were performed on 70 µg of protein extracts from C91/PL (HTLV-1-infected) lymphocytes transfected either with 75 nM of Gem siRNA or control siRNA. Membranes were probed with anti-Gem (1∶2,000), anti-Tax Tab 172 (1∶500) or anti-β-actin (1∶40,000) antibody. (B): Schematic representation of the protocol used for the cellular migration assays. C91/PL cells were transfected either with 75 nM of Gem siRNA or control siRNA. Twenty-four hours post-transfection, 5×10^5^ cells were collected and loaded on a 5 µm permeable Transwell filter. (C): Cell migration was quantified by flow cytometry. (D): Western blot analyses were performed on 70 µg of cellular extracts from C91/PL transfected either with 75 nM of Gem siRNA or control siRNA. Membranes were probed with anti-Gem (1∶2,000) or anti-β-actin (1∶40,000) antibody. *: significantly different, p = 0.018, Student's t-test.

To test this hypothesis, Gem siRNA or control siRNA were transfected into C91/PL cells. As expected, Gem expression dramatically decreased in Gem siRNA transfected cells but not in cells transfected with irrelevant siRNA (Figure 7A, lanes 6–8 vs. 2–4). As controls, Tax and actin western blots were performed and did not show any significant change (Figure 7A lower panels).

Using Transwell migration assays, we then measured the spontaneous migration of C91/PL after transfection of Gem siRNA or control siRNA (Figure 7B). A 33% decrease in migration was observed in C91/PL transfected with Gem siRNA (Figure 7C, p = 0.018, Student's t-test). As a control, Gem western blot was performed using cell extracts from cells transfected with control or Gem siRNA (Figure 7D). Altogether, these results demonstrate that Gem plays an essential role in the enhanced cellular migration of HTLV-1-infected cells.

### Gem expression increases cellular migration

**Figure 8:**
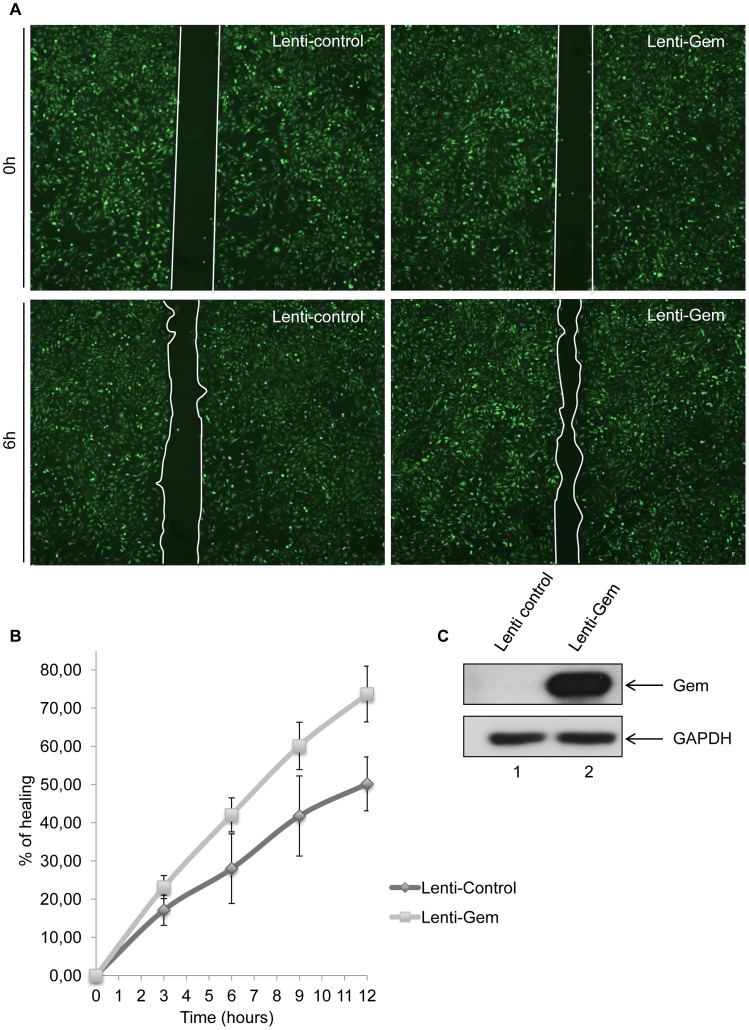
Gem expression is sufficient to increase cell motility. (A): HeLa cells were transduced with Lenti-control or Lenti-Tax viral particles. When cells reached 100% confluence, a wound was made in the cell monolayer and pictures were taken at 0 h and up to 12 h post-wounding. Measurements between the 2 fronts of migration were performed using image-J software (NIH, USA). (B): Percentage of healing during a 12 h kinetic. (C): Western blot analyses were performed on 70 µg of cellular extracts transduced by Lenti-control or Lenti-Tax viral particles. Membranes were probed with anti-Gem (1∶2,000) or anti-GAPDH (1∶1,000) antibody.

To test whether Gem is sufficient to increase cell motility, we then performed a wound-healing assay (Figure 8). Seventy-two hours post-transduction with control or Gem lentiviral particles, Hela cell monolayers were wounded and a first image was taken (time = 0 h). Of note, the presence of an IRES-GFP in the lentiviral vectors allows the monitoring of transduced (ie. GFP-positive cells) cells. Pictures were then taken after 3 h, 6 h, 9 h and 12 h, and distance between the 2 fronts of migration was measured (Figure 8A–B). Interestingly, after 12 h, Gem-expressing cells migrated 33% faster than cells transduced with the control lentiviral particles (Figure 8B). Of note, 100% healing would mean that the wound is totally closed. The difference between control and Gem expressing cells was visible at each time point (Figure 8B). As a control, Gem expression was determined by western blot (Figure 8C). These results demonstrate that Gem expression is sufficient to increase Hela cells spontaneous migration.

**Figure 9:**
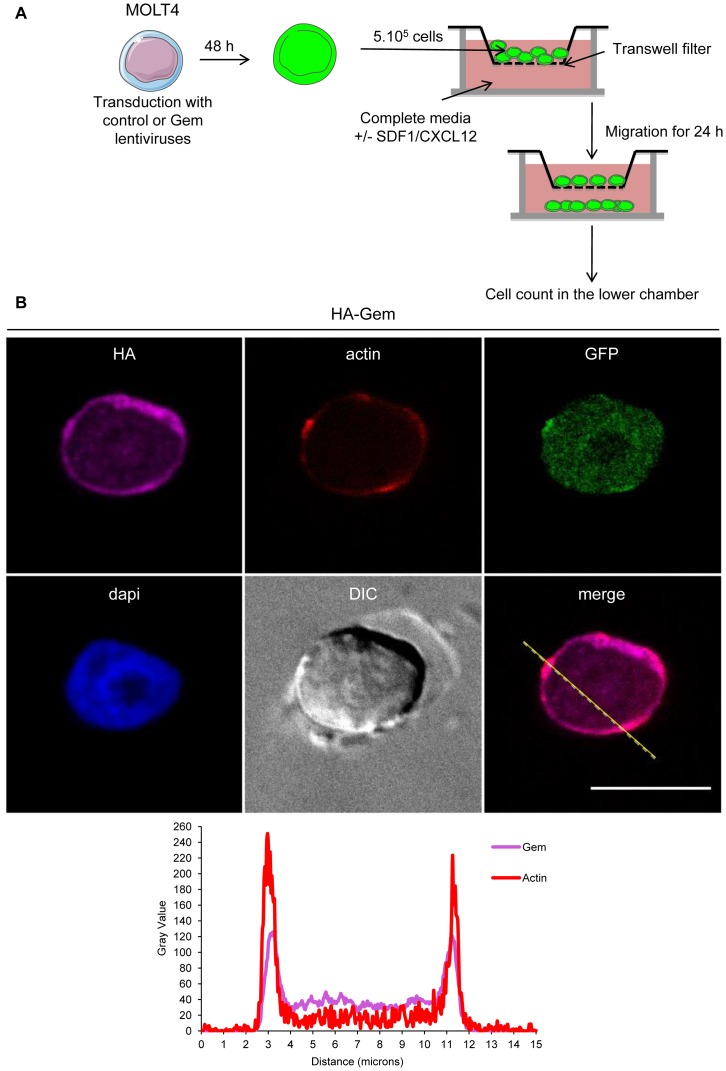
Gem expression is sufficient to increase chemokinesis and chemotaxis. (A): Schematic representation of the protocol used for the cellular migration assay. (B): MOLT4 cells were transduced with control or Gem viral particles. Forty-eight hours later, cells were processed as described above. Two-dimensional graphs representing pixel intensities (gray level) along a 15 micrometers line (see yellow line on the merged image) were plotted. Cyan line displays HA-Gem signal intensity and red line displays F-actin signal intensity (phalloïdin-rhodamine).

**Figure 10:**
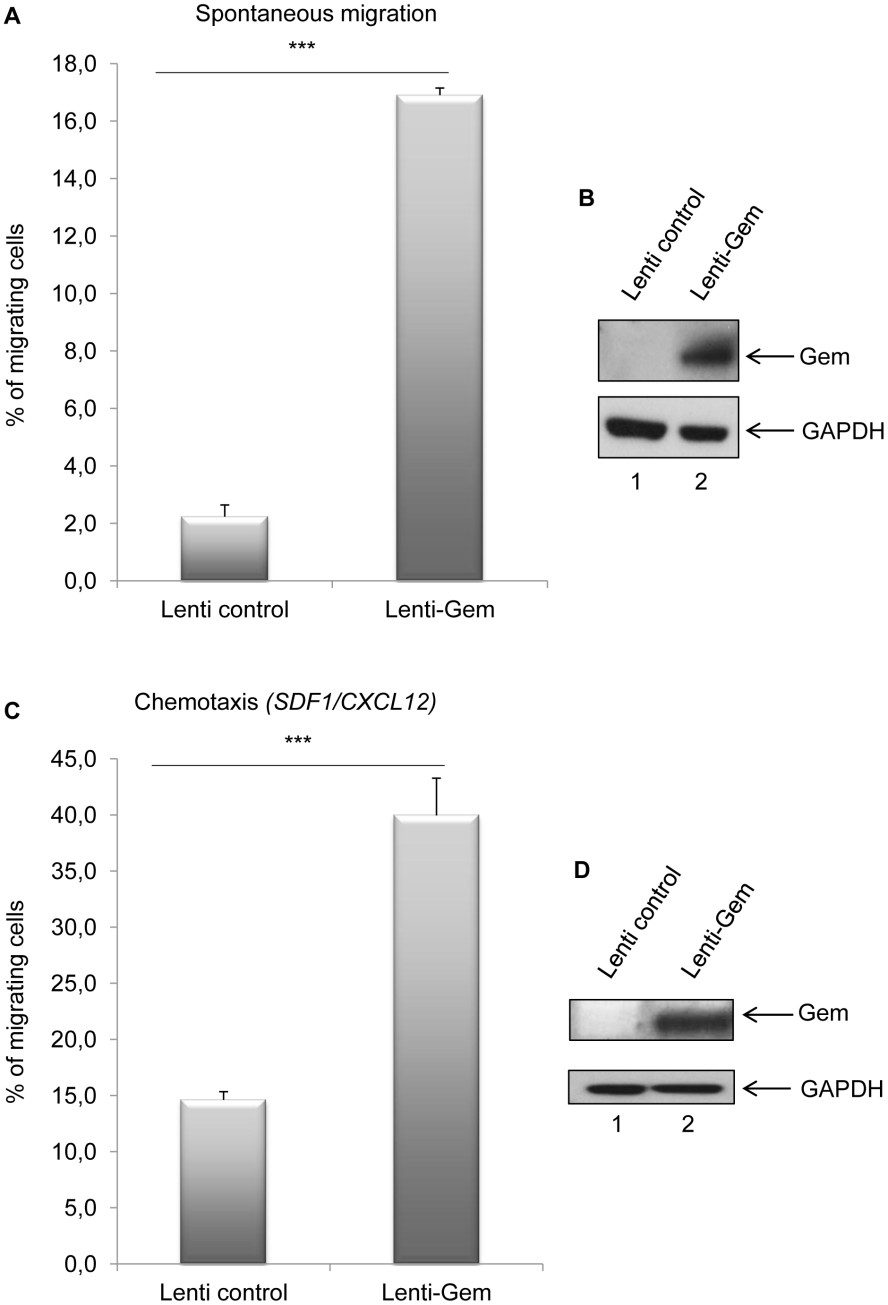
Gem expression is sufficient to increase chemokinesis and chemotaxis. (A, C): MOLT4 cells were transduced with Lenti-control or Lenti-GEM. Forty-eight hours later, 5.10^5^ cells were collected and loaded on a 5 µm permeable Transwell filter in absence or presence of SDF1/CXCL12 (150 ng/ml). Twenty-four hours later, cell migration was quantified by flow cytometry (flow cytometer Facscalibur4c+HTS (BD biosciences)). (B, D): Western blot analyses were performed on 70 µg of cellular extracts from MOLT4 cells transduced by Lenti-control or Lenti-GEM viral particles. Membranes were probed with anti-Gem (1∶2,000) or anti-GAPDH (1∶1,000) antibody. ***: significantly different, p<0.0001, Student's t-test.

Since T-cells are the primary targets of HTLV infection *in vivo*, we then assessed the role of Gem in chemokinesis (spontaneous cellular migration) and in chemotaxis (in presence of chemoattractant) after MOLT4 transduction with Gem or control lentiviral particles. The ability of cells to transfer from the upper chamber into the lower chamber of a Transwell permeable filter was measured in absence or presence of a chemoattactant (SDF1/CXCL12). Percentage of migrating cells was then quantified by flow cytometry (Figure 9A). We first assessed the localization of Gem in MOLT4 transduced cells and observed colocalization of Gem with cortical actin (Figure 9B). This result is consistent with results observed in Figures 3A, 4 and 5. An 8-fold increase in migration was observed in T-cells transduced with Gem lentiviral particles compared to controls (Figure 10A, p<0.0001, Student's t-test). In the presence of SDF1/CXCL12 (Figure 10C), 40% of Gem transduced MOLT4 cells migrated into the lower chamber, while only 15% of the MOLT4 cells transduced with the control lentiviral particles migrated (p<0.0001, Student's t-test). As a control, Gem expression was monitored by western blot (Figure 10B and 10D). These experiments demonstrate that Gem expression is sufficient to increase T-cell chemokinesis and chemotaxis.

### Gem is involved in conjugate formation

**Figure 11:**
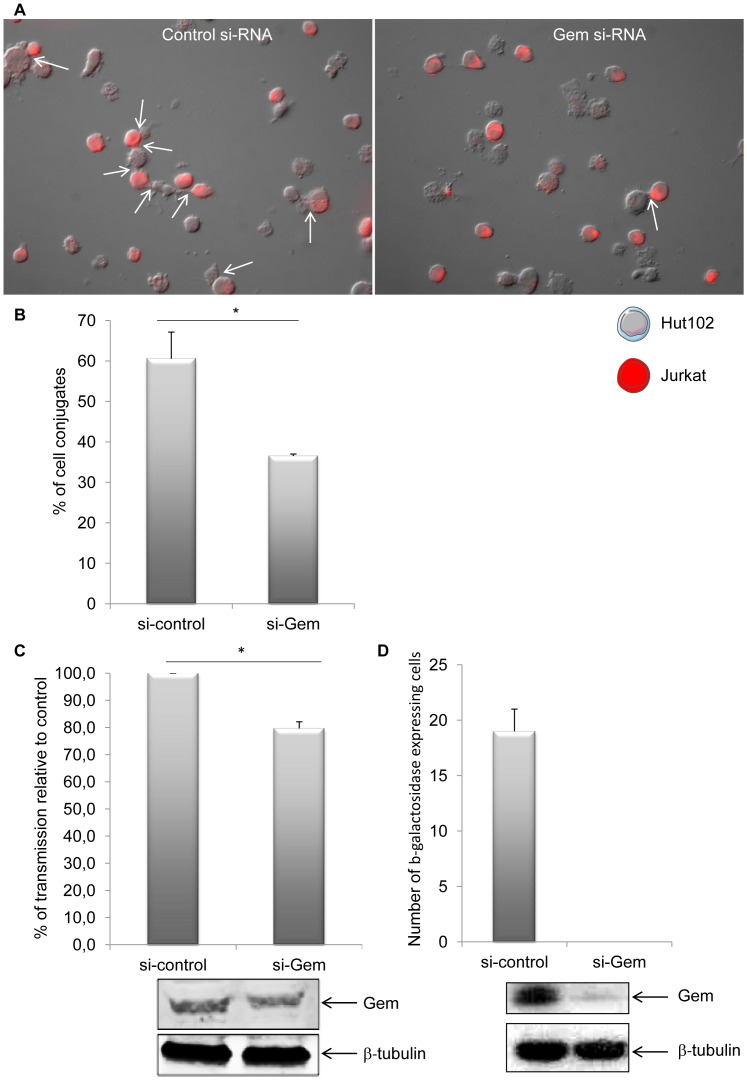
Gem is directly involved in cell-to-cell conjugates formation and virus transfer. (A): Jurkat T-cells were labeled with CellTracker Red CMPTX (577 nm, 2 µM, Invitrogen). HTLV-1-infected cells (Hut102) were transfected with (right panel) 75 nM of Gem or (left panel) control siRNA. Twenty-four hours later, Hut102 were mixed with Jurkat at a 1∶1 ratio on 0.01% poly-L-lysine (Sigma) treated Lab-Tek (Nunc) and incubated for 1 h at 37°C. Cells were visualized and signals acquired with an Axioimager Z1 microscope (Zeiss). White arrows represent the cell-to-cell conjugates. (B): Graph represents the results of 3 independent experiments. (C) Hut102 cells were transfected either with 75 nM of Gem siRNA or with control siRNA. Forty-eight hours later, those cells were cocultured with target Jurkat cells labelled with CellTracker Red CMPTX (577 nm, 2 µM, Invitrogen). After 20 min of co-culture, the percentage of gag p19 positive Jurkat cells was determined by flow cytometry. Graph represents the result of 3 independent experiments. (D) C91/PL cells were transfected with 75 nM control or Gem siRNA together with a GFP plasmid. Twenty-four hours later GFP positive cells were harvested by cell sorting. Productive viral infection was measured using BHK1E6 cells that carry the lacZ gene driven by the HTLV-1 LTR promoter. The graph represents the average and standard deviation of three independent wells. The level of Gem and β-tubulin in the GFP positive cells is shown as indicated. *: significantly different.

Efficient transmission of HTLV-1 from infected to uninfected T-cells relies on cell-to-cell contacts. Tax and p8 (cleavage product of p12) were previously shown to be involved in formation of cell-to-cell conjugates [14], [34]. In order to determine whether Gem plays a role in this process, Hut102 (HTLV-1-infected) were transfected with Gem siRNA or control siRNA before culturing them with Jurkat target cells that have been labeled with dye (Figure 11A). A 40% reduction (p = 0.05, Mann-Whitney test) in the number of T-cell conjugates (Figure 11A, white arrows) was observed when Jurkat cells were incubated with Hut102 cells transfected with Gem siRNA (Figure 11B). Similar results were obtained with MT2 cells (data not shown). These results indicate that Gem is directly involved in the formation of T-cell conjugates and is, therefore, likely to play a role in viral transmission from infected cells to target cells.

### Gem is involved in viral transmission but not in viral production

Finally, to assess whether Gem could be involved in viral transmission, we performed a cell-to-cell viral transfer experiment. Hut102 cells were transfected with Gem siRNA or control siRNA. Forty-eight hours post transfection they were co-cultured for a short period of time (20 minutes) with Jurkat target cells that have been previously labeled with dye. We then monitored by flow cytometry the level of intracellular gag p19 in the Jurkat cells. p19 signal monitors viral transfer from infected cells toward target cells (Figure 11C). These results demonstrate that a reduction in Gem expression directly impacts viral transmission, decreasing the number of cells infected by 20% (p = 0,02, Student's t-test). This result is consistent with the 40% reduction in conjugate formation observed in figure 11B. As control, Gem and beta-tubulin levels were monitored by western blot analysis. Of note, a 22% decrease in Gem level expression was observed in cell transfected with Gem siRNA (Figure 11C lower panel).

To determine if the decrease in viral transfer impacted productive infection, we transfected C91/PL cells with control or Gem siRNA and a plasmid expressing GFP. Twenty-four hours after transfection, GFP positive cells were harvested by cell sorting of the transfected cultures. These cells were then co-cultured for one hour with a reporter cell line BHK1E6, containing the lacZ gene driven by the HTLV-1-LTR promoter. Cells transfected with control siRNA were capable of transmitting virus to target cells resulting in expression of β-galactosidase. In contrast, no β-galactosidase positive cells were measured when Gem protein expression was knocked down (Figure 11D, graph). As a control Gem expression was monitored by western-blot (Figure 11D, lower panel).

To be sure that the decrease in viral transmission was not due to a decrease in viral production and release following siRNA transfection, Gag p19 was monitored by ELISA (Zeptometrix Corporation) from 24 h to 72 h in the cell culture supernatant of HTLV-1 cells treated with Gem siRNA or control siRNA (Figure S1). We did not observe any significant difference in viral production in cells treated with Gem siRNA. This confirms that the decrease in viral transmission seen above is not due to a decrease in viral production but to the direct role of Gem in viral transfer.

Altogether, these results indicate that Gem is not only involved in the formation of T-cell conjugates but also in viral transmission from infected cells to target cells.

## Discussion

In contrast to HIV-1, HTLV-1-infected cells produce few free viral particles [10]. Indeed, efficient transmission of the virus from an infected cell to an uninfected T-lymphocyte relies mostly on cell-to-cell contacts via the formation of a virological synapse and/or of a viral biofilm-like structure [12]–[15]. In both models, Tax plays an important role. Thus, one can assume that blocking the formation of cell conjugates would have a major impact on cell transmission and proviral load evolution.

We previously identified a set of genes that are up-regulated in T-cells transduced by HTLV-1 Tax encoding lentivirus [17]. Here, we performed a retrospective analysis, focusing our attention on genes that could potentially be involved in HTLV viral transmission, and identified *gem*. Interestingly, data mining allowed us to retrieve previous reports which also listed *gem mRNA* among the genes that are up-regulated gene in Tax-transformed lymphocytes (Tesi), Tax-inducible JPX-9 cells, HTLV-1-transformed (C8166 and Hut102) or -immortalized cells (Bes, ACH.WT and Champ) [17], [25]–[27]. Our results also demonstrate that Gem is strongly expressed at the protein level following Tax expression in T-lymphocytes and in all HTLV-1-infected cell lines tested. The fact that Gem expression is directly linked to Tax expression was confirmed by the reporter gene and Chip assays, demonstrating that together with CREB and CBP, Tax activates transcription from the *gem* promoter through a CRE sequence.

Small GTPases of the Rho family are pivotal regulators of several aspects of cell behavior, such as cell motility, cell proliferation and apoptosis. They play a central role in many motile responses that involve the actin cytoskeleton and/or microtubule network, from neurite extension to phagocytosis and cancer-cell invasion [35]. Our results show that Gem strongly colocalizes with F-actin and are consistent with its direct involvement in actin cytoskeleton dynamic. Polymerization of actin soluble units in filaments is a major mechanism for the cell movement [31]. Interestingly, *Kress et al.*, showed that fascin, a protein that stabilizes filamentous actin and concentrates in cellular protrusions, such as filopodia, during cell migration, is overexpressed following Tax expression. This protein is overexpressed in HTLV-1-infected cell lines and involved in HTLV-1 cellular invasion [25]. Moreover, *Varrin-doyer et al.*, also demonstrated that the collapsing response mediator protein 2 (CRMP2) was overexpressed in HTLV-1-infected T-lymphocytes *in vitro*, showed that CRMP2 was involved in migration of these cell clones and demonstrated that Tax was partly involved both in modulation of CRMP2 level and lymphocyte migratory rate. In addition, CRMP2 remodels T-lymphocyte microtubule cytoskeleton and partially co-localized with Tax at the cell-to-cell contact point in HTLV-1-infected cells [32]. We did not observe such co-localization between Gem, Tax and actin within the cells. However, expression of Gem either directly or through Tax induction was sufficient to increase lymphocyte migratory rates, and Gem suppression in HTLV-1-infected cells severely reduced cellular migration. These results demonstrate a direct role of Gem in HTLV-1-infected lymphocytes migration and it would now be interesting to define whether their shape was also modified. Interestingly, it has been demonstrated that HIV-1 is also able to modify actin dynamics in order to promote HIV infection. In fact, *Yoder et al.*, demonstrated recently that during HIV infection the actin cytoskeleton in resting T-cells is a post-entry barrier for HIV-1 [36], [37]. However, the virus has developed a strategy to overcome this restriction. Binding of the gp120 envelope glycoprotein to the CXCR4 chemokine co-receptor induces activation of a cellular protein named cofilin. Cofilin is a critical factor for depolymerizing the cortical actin filaments (F-actin), therefore allowing migration of the viral material into the cell.

Gem is a negative regulator of the ROCK-I (ROKβ) Rho kinase. When overexpressed, Gem inhibits ROCK I-induced neurite retraction and ROCK-mediated phosphorylation of myosin light chain (MLC) and myosin light chain phosphatase (MLCP) [30]. Interestingly, CRMP2 is phosphorylated by another Rho kinase: ROCK-II (ROKα). CRMP2 normally promotes axon outgrowth, possibly through its ability to promote microtubule assembly. It has been shown that CRMP-2 phosphorylation is involved in the regulation of the neurons growth cone morphology [38]. Fascin is also a target of Rho-kinases. Rho activity modulates the interaction of Fascin with the p-Lin-11/Isl-1/Mec-3 kinase 1 and 2 (LIMK1/2), which allow the recruitment of this complex to actin filaments. This interaction modulates actin filopodia dynamics and promotes its stabilization [39]. Thus, CRMP2 and Fascin have been proposed to function down-stream of Rho kinases [35], [39], whereas Gem is an upstream negative regulator of ROCK-I Rho kinase [30]. Altogether, these results suggest that Fascin, CRMP-2 and Gem are different actors that play a role in the regulation of HTLV-1-infected cell motility and are therefore involved in viral transmission. However, our results show that Gem could have additional functions during HTLV-1 infection. First, it is involved in the formation of conjugates between infected and uninfected T-lymphocytes. Of note, Tax and p8 were also previously shown to be involved in the formation of cell-to-cell conjugates that involve cytoskeleton reorganization [14], [31]. In the HIV-1 situation, the virus is also transmitted through the production of free viral particles by the infected cells [40]. In contrast, HTLV-1 transmission occurs mainly through cell-to-cell contacts [11]–[13],[16]. Thus, we hypothesize that the enhanced cell migration upon HTLV-1 infection increases the probability of encountering and infecting new target cells through the creation of cell-to-cell contacts.

Thus our results indicate that HTLV-1-infected cells, which express Gem, are likely to migrate faster and to form more conjugates with target T-cells and thus to transmit more efficiently the virus. It would now be of interest to assess simultaneously the relative contribution of Fascin, CRMP-2 and Gem in HTLV-1-infected cells motility and viral transmission.

In conclusion, our results demonstrate that HTLV-1 Tax protein promotes Gem expression *in vitro* and *in vivo*. As a consequence, Gem modifies cell morphology and increases migration of HTLV-1-infected cells as well as formation of cell-to-cell conjugates.

Finally, we demonstrate that Gem plays an important role in cell-to-cell viral transmission.

## Materials and Methods

### Cell culture

293T, HeLa, Jurkat and MOLT4 cells were obtained from ATCC. HTLV-1-infected cell lines and JPX-9 cell line were obtained from Dr Antoine Gessain (Pasteur Institute, Paris). 293T and HeLa cells were cultured in DMEM-GLUTAMAX-I (Gibco, Invitrogen) complemented with 10% fetal bovine serum (FBS) (Gibco, Invitrogen) and antibiotics (penicillin-streptomycin, PAA). MOLT4, Jurkat, JPX-9 and HTLV-1-infected cell lines (C8166, C91/PL, Hut102 and MT2) were cultured in RPMI-GLUTAMAX-I (Gibco, Invitrogen), complemented with 10% fetal bovine serum (FBS) (Gibco, Invitrogen), and antibiotics (PAA). Cells were maintained at 37°C in 5% CO_2_. Tax expression was induced by adding 120 µM of ZnCl_2_ in the culture media of JPX-9 cells [41].

### Lentiviral particles production and transduction experiments

Production of Lenti-IRES-GFP (named “Lenti-control” thereafter), Lenti-Tax-IRES-GFP (named “Lenti-Tax” thereafter) and Lenti-HA-Gem-IRES-GFP (named “Lenti-Gem” thereafter) lentiviral particles as well as cell transduction procedures were performed as previously described [17]. Stocks of lentiviral particles were stored at −80°C.

### Plasmids

Gem cDNA was amplified from pMT2T-Gem vector [42] and cloned either in frame with a HA tag into the pSG5M vector [43] or into the pSDM101 vector [17]. Tax-1 cDNA was amplified from pSG5M-Tax-1 vector [43] and cloned in frame with a Flag tag into the pSG5M vector.

### RT-PCR

Seventy-two hours post-transduction, total RNAs were extracted from 293T cells using the RNeasy mini kit (Qiagen). To avoid DNA carryover, RNA samples were treated with DNase I RNase-free DNAs set (Qiagen). Five hundred nanograms of total RNA were used as a matrix for RT-PCR using the one step RT-PCR kit (Qiagen). PCRs were performed with an annealing temperature of 52°C for Gem primers (Fwd: [5′-GAACTAGGCTCATCAGAATCGTGAC-3′]; Rev: [5′-GAGCTGTGACATACAAGGGTCAACC-3′]) or of 59°C for GAPDH primers ([17]).

### Chromatin immunoprecipitations (Chip) assay

ChIP assay was carried out using 10 µg of antibody to Tax (Tab 172, NIH), CREB or CBP (Santa Cruz biotechnologies) antibodies as described previously [44], [45]. C8166 cells (5×10^7^) were cross-linked and sheared by sonication to get 200- to 800-bp DNA fragments. Chromatin extracts were pre-cleared with salmon sperm DNA and magnetic protein G beads (Sigma). Supernatants were diluted 10-fold with ChIP dilution buffer, and appropriate antibodies were added and incubated overnight at 4°C. Immune complexes were then collected by addition of magnetic protein-G beads and washed stepwise. Cross-linking was reversed and DNA was purified by proteinase K (Sigma) treatment, phenol extraction, and ethanol precipitation. PCR was performed using primers specific for the HTLV-1 LTR (nucleotides −160 to −139 [5′-CCACAGGCGGGAGGCGGCAGAA-3′] and nucleotides −102 to −79 [5′-TCATAAGCTCAGACCTCCGGGAAG-3′]) or Gem promoter (nucleotides −759 to −738 [5′-GAGATGCTGCTGATTGGATGC-3′] and nucleotides −136 to −115 [5′-CCTCTGCAGCAAACTCGGAGT-3′]).

### Immunoblot analysis

Lentivirus-transduced cells, HTLV-1-infected or siRNA-transfected cells were collected and washed with PBS. Proteins were extracted using WCE buffer (50 mM Tris-HCl pH 8, 120 mM NaCl, 5 mM EDTA, 0.5% NP40, 1 mM PMSF, 1 mM DTT, 50 mM NaF, 0.2 mM Na_3_VO_4_) in the presence of protease inhibitors (Complete-EDTA-free, Roche). Protein amounts were quantified using the Bradford reagent assay (Biorad) and 40–70 µg were loaded into 4–12% NU-PAGE gels (Invitrogen). Following electrophoresis and protein transfer onto PVDF membranes, membranes were blocked for 1 h in a 5% milk/PBS-Tween 0,05% solution and incubated overnight with primary antibody (1∶2000 anti-Gem ([42]), 1∶400 anti-p24 (TP-7 clone, Zeptometrix), 1∶4,000 anti-Flag M2 (Sigma), 1∶40,000 anti-β-actin (AC74, Sigma), anti-GAPDH (Novus, NB300-322), 1∶1000) and anti-β-tubulin (Santa Cruz, H-235). The next day, membranes were washed and incubated either with anti-rabbit or with anti-mouse horseradish peroxidase-conjugated secondary antibodies (GE Healthcare). Membranes were then developed using the SuperSignal West Pico (Pierce/Thermoscientific) or ECL plus kit (GE Healthcare).

### Immunofluorescence

HeLa cells were transfected with 200 ng of pSG5M-HA-Gem or pSG5M backbone plasmid using Effectene reagent (Qiagen) following manufacturer's instructions. Peripheral Blood Lymphocytes (PBLs) were electroporated with 5 µg of pSG5M-HA-Gem. Gem transduced MOLT4 and Gem electroporated PBLs were seeded on 0.01% poly-L-lysine (Sigma) coated coverslip before fixation in 4% PFA solution (Sigma). Twenty-four (Hela) or 48 hours later (PBLs), cells were fixed in 4% PFA solution (Sigma) for 20 min, neutralized with NH_4_Cl for 10 min and permeabilized with 0.5% Triton X-100 (Sigma) for 5 min. Following PBS washes, cells were incubated with a 5% PBS/milk solution then with anti-HA (Sigma, H6908, 1∶150 dilution in 5% PBS/milk) or anti-Flag M2 (Sigma) antibody (1∶1000 dilution in 5% PBS/milk) for 1 h at room temperature. F-actin was stained with Rhodamine Phalloidin toxin (R415 300U, Life Technologies). Samples were then incubated with FITC-conjugated goat anti-IgG rabbit (Vector Laboratories) (1∶100) or with CY3-conjugated goat anti-IgG mouse (Amersham Biosciences) at a 1/1000 dilution in 5% PBS/milk for 1 h at room temperature. For the immunofluorescence of Gem performed in transduced Molt4, samples were incubated with DyLight 649-conjugated sheep anti-IgG rabbit (STAR36D649 AbD serotec). Nucleic acids were stained with DAPI-containing mounting medium (DAPI Fluormount G, Southern biotech) and cells were visualized and signals acquired with a spectral Leica SP5 confocal microscope. Images analyses were performed with the Fiji software [46]. We used Plot Profile tool in Image-J software to create a plot of intensity values across features in our images.

### Luciferase assays

Forty-eight hours post-transduction with control or Tax lentiviral particles (MOI = 1), 293T cells were transiently transfected, either with a Gem-luc or a Delta-CRE-Gem-luc (500 ng) plasmid [28] using Polyfect reagent (Qiagen). Transfections were carried out in the presence of a phRG-TK vector (10 ng) in order to normalize the results for transfection efficiency. Reporter activities were assayed 24 h post-transfection using the dual-luciferase reporter assay system (Promega) as previously described [47]. Luciferase assays were performed with the GLOMAX microplate luminometer (Promega).

### Wound healing assay

One hundred twenty thousand Hela cells were seeded in 12-well plates. Twenty-four hours later, cells were transduced with either control (Lenti-IRES-GFP) or HA-Gem lentiviral particles (MOI = 1). Seventy hours post-transduction, a wound was performed in the cell monolayer when cells reached confluence. Pictures were then taken every 3 hours up to 12 h post-wounding with a Carl Zeiss Axiovert 135. Distances between the 2 fronts of migration were measured using Image-J software (NIH, USA).

### Cellular migration assay

MOLT4 or Jurkat cell lines were transduced with control (Lenti-IRES-GFP) or HA-Gem lentiviral particles (MOI = 5). Forty-eight hours later, 5×10^5^ transduced cells were loaded in the upper chamber of 5 µm polycarbonate transwell filters (Corning). Complete RPMI medium containing or not SDF1/CXCL12 chemo-attractant (150 ng/ml) was added in the lower chamber. Twenty-four hours later, quantification of the number of migrating cells was performed using TruCount flow tubes (BD Biosciences) and flow cytometer Facscalibur4c+HTS (BD biosciences).

HTLV-1-infected cells (C91/PL, Hut102) or non-infected control cells (Jurkat, MOLT4) were transfected with 75 nM of Gem siRNA (ON-TARGETplus smart Pool Gem siRNA L-008717-00-0020, Thermo Scientific) or siRNA control (ON-TARGETplus Non-targeting Pool, Thermo Scientific) using Hiperfect reagent (Qiagen). 5×10^5^ transfected cells were loaded in the upper chamber of 5 µm polycarbonate transwell filters (Corning) whereas complete RPMI medium was added in the lower chamber. Quantification was performed as described above.

### Conjugate formation

Jurkat T-cells were stained (CellTracker Red CMPTX 577 nm, 2 µM, Invitrogen) in RPMI-GLUTAMAX-I (Gibco, Invitrogen) in the absence of FBS for 30 min at 37°C. Cells were then washed and incubated in RPMI complete media for 45 min. HTLV-1-infected cell lines (C91/PL, Hut102 and MT-2) were transfected with 75 nM of Gem siRNA or control siRNA (Thermo Scientific) using Hiperfect reagent (Qiagen). After twenty-four hours, these cells were mixed with pre-stained Jurkat-T cells (ratio 1∶1) on 0.01% poly-L-lysine (Sigma) treated Lab-Tek (Nunc) and incubated for 1 h at 37°C. Cells were then fixed (4% Formalin, Sigma) for 20 min at room temperature and mounted in DAPI Fluormount G (Southern biotech). Signals were acquired with an Axioimager Z1 microscope (Zeiss). At least 15 pictures were taken for each condition and more than 1000 HTLV-1-infected cells were counted per condition. Image analyses were performed with the Image-J software. Data are presented as the mean from 3 independent experiments.

### Analysis of cell-to-cell HTLV-1 transfer and infection

Hut-102 cells were transfected either with Gem siRNA (ON-TARGETplus smart Pool Gem siRNA L-008717-00-0020, Thermo Scientific) or with control siRNA (ON-TARGETplus Nontargeting Pool, Thermo Scientific). Forty-eight hours post-transfection, these cells were co-cultured with CellTracker Red (2 µM, Invitrogen) CMPTX-labelled Jurkat target cells for 20 min. Donor and target cells were mixed at a concentration of 10^5^ cells/mL each. Cells were then fixed with 4% paraformaldehyde, permeabilized with triton 0,05%, and stained for intracellular p19 expression (Zeptometrix Corporation). Analysis was performed with a FACSCalibur flow cytometer (BD biosciences).

Transfer of infectious virus was measured using BHK1E6 cells that contain a lacZ reporter gene driven by the HTLV-1 LTR promoter [48]. C91/PL cells were transfected with 75 nM control or Gem siRNA and 0.05 µg of pMax-GFP plasmid using the Human T cell Nucleofector Kit, program O-017 (Lonza, Basel, Switzerland) as described by the manufacturer. Twenty-four hours after transfection, GFP positive cells were harvested by cell sorting on a FACSAria III cell sorter (BD Biosciences, San Jose, CA). GFP^+^ cells were rested for one hour in complete media and then 1×10^5^ cells were co-cultured with a monolayer of 1×10^5^ BHK1E6 cells, in a 6-well poly-lysine coated plate. After one hour of co-culture, GFP^+^ cells were removed, BHK1E6 cells washed three times with phosphate buffered saline (PBS) and cultured in fresh media for 24 hours. The next day, monolayers were washed twice with PBS and assayed using a β-galactosidase Staining Kit according to manufacturer's directions (Active Motif, Carlsbad, CA). After staining, β-galactosidase expressing cells were counted by brightfield microscopy.

### Statistical analyses

Analyses were performed in GraphPad Prism software Version 5.0b. When considering spontaneous migration of lymphocytes, chemotaxis to SDF-1 or cell-to-cell transfer, 1-tailed Student's *t*-tests and one-way ANOVA (with Dunnet post-hoc test) were used to compare the mean between 2 and 3 groups respectively. For quantification of wound healing assay or conjugate formation, Mann-Whitney test were performed. For quantification of promoter activation, i.e. luciferase assays, repeated one-way ANOVA (with Tukey post-hoc test) were used. *P* values less than .05 were considered significant.

### Measure of p19 in the cell-culture supernatant

One million HTLV-1-infected cells (C91/PL) were transfected with 75 nM of Gem siRNA (ON-TARGETplus smart Pool Gem siRNA L-008717-00-0020, Thermo Scientific) or control siRNA (ON-TARGETplus Nontargeting Pool, Thermo Scientific) using Hiperfect reagent (Qiagen). Twenty-four, forty-eight or seventy-two hours post-transfection, supernatants were collected and centrifuged at 2 000 rpm for 3 min. Supernatants were diluted in RPMI complete media (1∶100) and p19 levels were assessed using the RETROtek HTLV p19 Antigen ELISA kit (Zeptometrix Corporation) following manufacturer instructions.

## Supporting Information

Figure S1 **Gem expression does not alter p19 gag release in the cell culture supernatant.** C91/PL (HTLV-1 positive) cells were transfected with 75 nM of Gem or control siRNA. Cell culture supernatants were collected twenty-four, forty-eight or seventy-two hours post transfection, and p19 gag levels were assessed using RETROtek HTLV p19 Antigen ELISA kit.
(TIF)

Table S1 **Retrospective database analysis on HTLV gene expression profiles of Tax expressing lymphocytes, HTLV-1-infected cells lines or HTLV-1 immortalized cells.** We focused our attention on publications involving gem/kir (italic). Affymetrix probes set number are presented in this table.
(TIF)
