# Supplementary figures and images for: Gem-Induced Cytoskeleton Remodeling Increases Cellular Migration of HTLV-1-Infected Cells, Formation of Infected-to-Target T-Cell Conjugates and Viral Transmission
Source: PLoS Pathog. 2014 Feb 27;10(2):e1003917. doi: 10.1371/journal.ppat.1003917 (PMC3937318; doi:10.1371/journal.ppat.1003917)

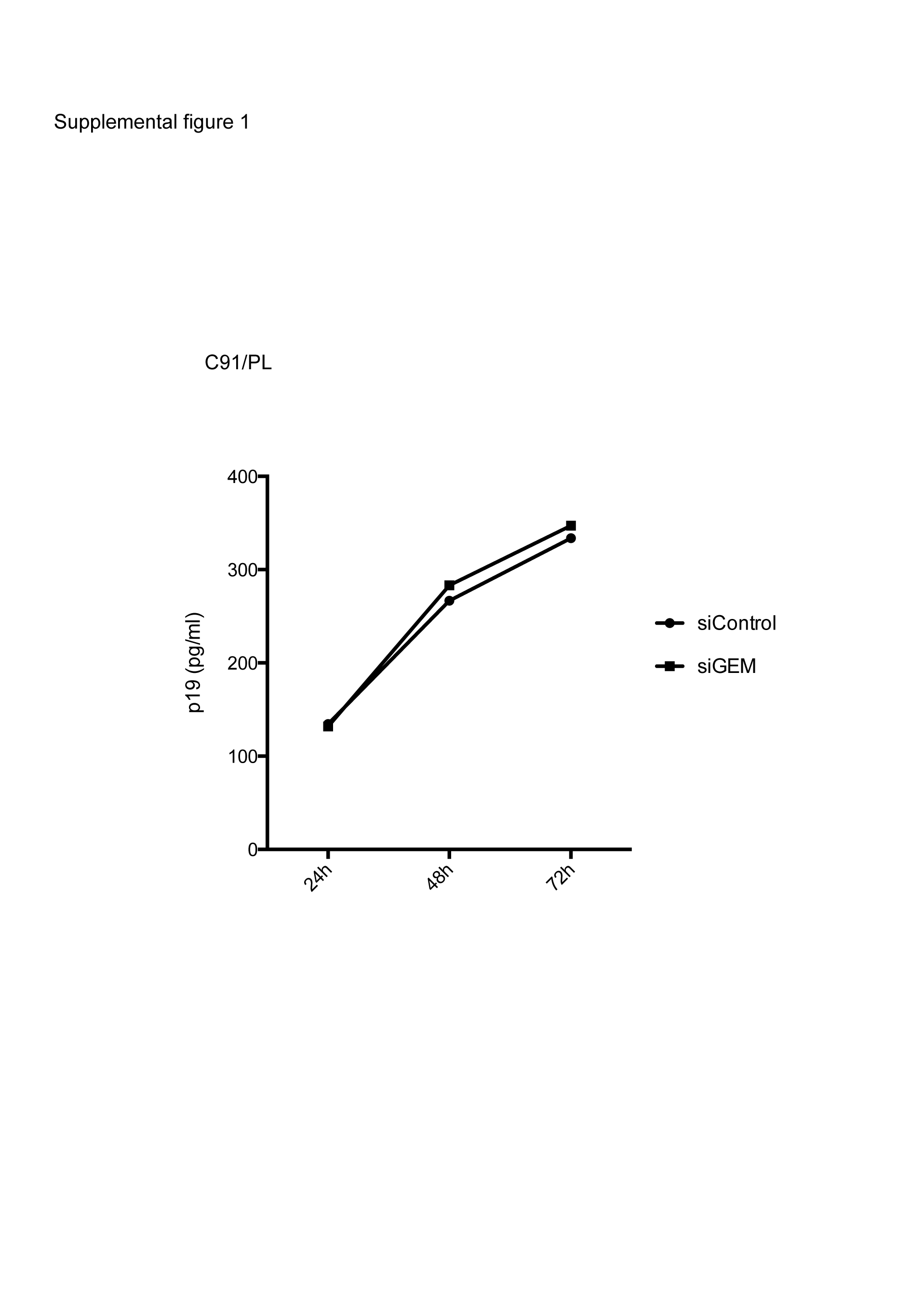

Supplement: Figure S1 — Gem expression does not alter p19 gag release in the cell culture supernatant. C91/PL (HTLV-1 positive) cells were transfected with 75 nM of Gem or control siRNA. Cell culture supernatants were collected twenty-four, forty-eight or seventy-two hours post transfection, and p19 gag levels were assessed using RETROtek HTLV p19 Antigen ELISA kit. (TIF) [file ppat.1003917.s001.tif]

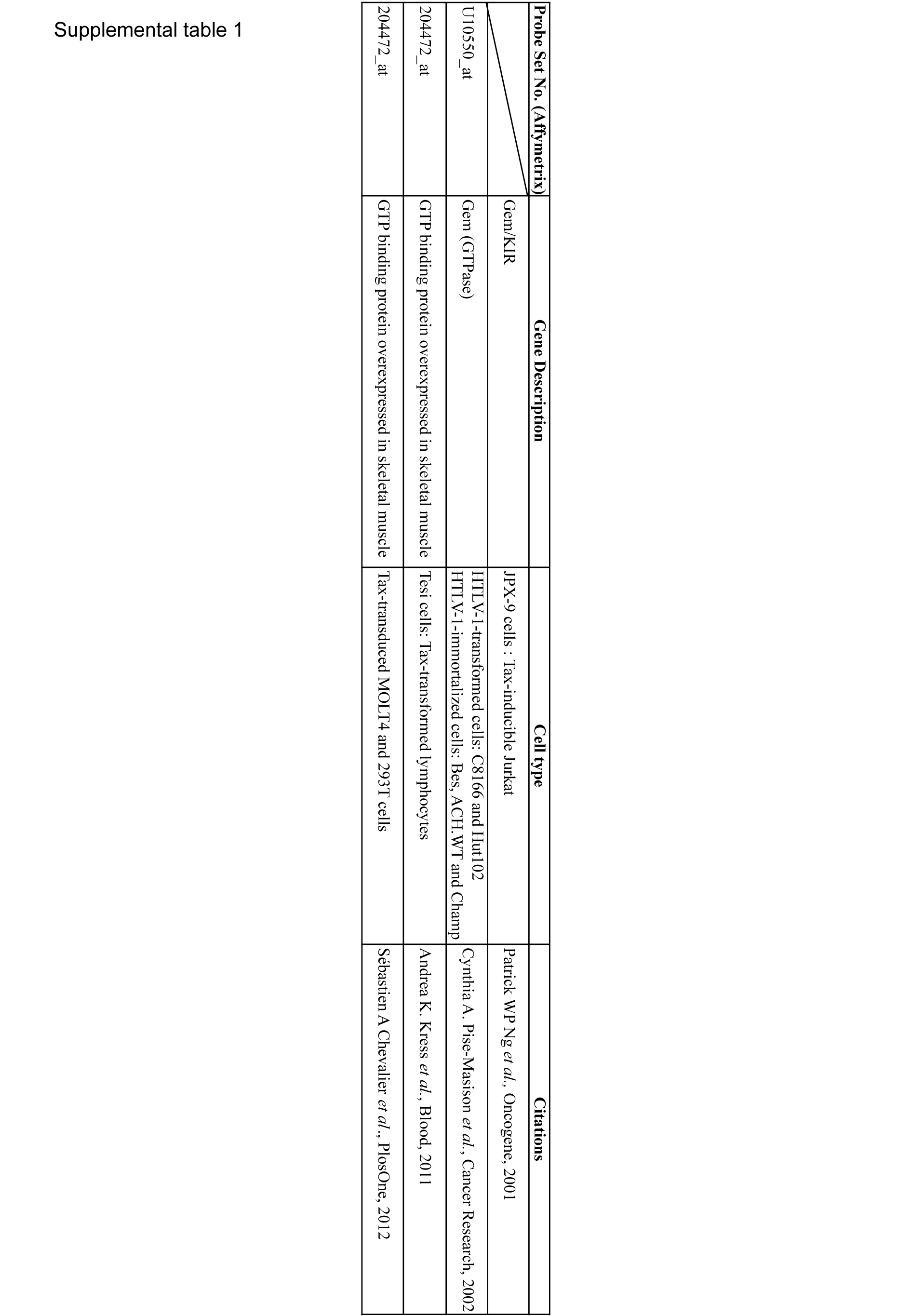

Supplement: Table S1 — Retrospective database analysis on HTLV gene expression profiles of Tax expressing lymphocytes, HTLV-1-infected cells lines or HTLV-1 immortalized cells. We focused our attention on publications involving gem/kir (italic). Affymetrix probes set number are presented in this table. (TIF) [file ppat.1003917.s002.tif]
